# Untargeted metabolomics and antioxidant responses reveal tissue- and species-specific metabolic profiles in three *Epimedium* species

**DOI:** 10.3389/fpls.2026.1942146

**Published:** 2026-10-13

**Authors:** Yu Zhang, Hong Zhang, Yuru Zhang, Di Wang, Xue Meng, Juan Chen

**Affiliations:** Institute of Chinese Materia Medica, Shaanxi Provincial Academy of Traditional Chinese Medicine, Xi’an, Shaanxi, China

**Keywords:** antioxidant activities, Epimedium species, metabolomic analysis, phenylpropanoid biosynthesis, secondary metabolic pathways

## Abstract

**Objectives:**

This study systematically analyzed the antioxidant responses, untargeted metabolomic profiles, and secondary metabolic pathways of three representative *Epimedium* species.

**Methods:**

Meteorological data (atmospheric pressure, temperature, relative humidity, and precipitation) were recorded from July 2022 to July 2025 to provide environmental background context. Antioxidant indexes including H_2_O_2_ and O_2_^-^ levels, as well as CAT, POD and SOD activities in different plant tissues were determined. Untargeted metabolomics was performed to explore metabolic differences. PCA, differential metabolite analysis, KEGG and MSEA enrichment were conducted to screen key metabolic pathways, and correlation analysis was employed to explore exploratory associations between antioxidant enzyme activities and secondary metabolite abundances.

**Results:**

The levels of H_2_O_2_ and O_2_^-^ were generally higher in roots than in stems and leaves. CAT activity showed no significant differences among tissues or species, whereas POD and SOD activities presented distinct tissue-specific and species-specific patterns. A total of 2730 MS features were detected. PCA and differential metabolite analysis revealed remarkable metabolic separation and diverse metabolite changes among the three *Epimedium* species. KEGG and MSEA enrichment indicated that differential metabolites were mainly involved in phenylpropanoid biosynthesis, flavonoid biosynthesis, galactose metabolism and so on. 46 MS features were putatively annotated at the compound level (MSI Level 2 or 3) via MS/MS fragment interpretation and literature evidence. Correlation analysis was performed to explore the associations between antioxidant enzymes and key secondary metabolites.

**Conclusions:**

The three *Epimedium* species exhibit distinct antioxidant characteristics and metabolic profiles under the local meteorological environment. Tissue-specific and species-specific differences are mainly concentrated in phenylpropanoid and flavonoid metabolism, with potential associations observed between these metabolites and the antioxidant system. These results provide descriptive insights into the physiological characteristics and quality formation of *Epimedium* species.

## 1 Introduction

The genus *Epimedium*, the largest herbaceous genus in the Berberidaceae family, has its center of diversity and distribution in China. The earliest record of *Epimedium* dates back more than 2, 000 years to *Shennong Bencao Jing* (Shennong’s Classic of Materia Medica). Later, during the Ming Dynasty, Li Shizhen documented *Epimedium* as a medium-grade herb in *Bencao Gangmu* (Compendium of Materia Medica) (Seyedi et al., 2023). The Chinese Pharmacopoeia encompasses five *Epimedium* species, namely *Epimedium wushanense* T.S. Ying, *Epimedium sagittatum* Maxim., *Epimedium brevicornu* Maxim., *Epimedium koreanum* Nakai, and *Epimedium pubescens* Maxim. To date, more than 379 chemical constituents have been reported in *Epimedium* species, including flavonoids, organic acids, lignans, terpenoids, alkaloids, and dihydrophenanthrene derivatives. Among these, flavonoids-including icariin, icariside I, baohuoside I, icaritin, and epimedin a, b, and c-have been recognized as the principal phytochemical and pharmacologically active constituents (Zhang et al., 2023). *Epimedium* has been used alone or in combination with other herbs to treat various conditions, including rheumatoid arthritis, cancer, osteoporosis, sexual dysfunction, and neurological disorders (Zhang et al., 2022). Published reviews have primarily concentrated on the active components, pharmacology, clinical applications and quality control of *Epimedium* species (Zhang et al., 2023; Wang S. et al., 2025). Existing metabolomics studies on *Epimedium* plants have mainly concentrated on pharmacodynamic investigations; the few available studies have only addressed flavonoid accumulation in floral tissues and component accumulation under UV-B radiation (Qin et al., 2022; Li et al., 2024; Liu et al., 2023). Therefore, there is a need for metabolomic studies covering multiple tissues of *Epimedium* plants to identify differential metabolites.

Additionally, plants must adapt to a variety of unfavorable conditions, such as elevated temperatures, air pollution, drought, salinity, heavy metal contamination, and pathogen attack. Under such conditions, plants produce increased amounts of reactive oxygen, nitrogen, and sulfur species (ROS/RNS/RSS), whose concentrations must be tightly regulated (Mukherjee and Corpas, 2023). The enzymatic antioxidant system and associated metabolites modulate these reactive species, preventing damage to cellular components while enabling their participation in signaling events. Through post-translational modifications (PTMs) of proteins, reactive species function as signaling molecules that modulate plant responses to environmental cues (Jiménez et al., 2025). ROS, including hydrogen peroxide (H_2_O_2_) and superoxide anion (O_2_^-^) are produced upon reduction of molecular oxygen in plants (Thorpe et al., 2013). These reactive species can damage proteins and lipids, leading to cellular injury and death (Nouman et al., 2014). Under optimal plant growth and development conditions, reactive oxygen species (ROS) accumulation within organelles remains at an extremely low basal level. Plant cells maintain ROS detoxification and cellular homeostasis through two distinct ROS-scavenging systems: enzymatic antioxidant systems and non-enzymatic antioxidant pathways. The canonical enzymatic antioxidant enzymes include superoxide dismutase (SOD), ascorbate peroxidase (APX), catalase (CAT), peroxiredoxin (PRX), dehydroascorbate reductase (DHAR), glutathione peroxidase (GPX), glutathione reductase (GR), glutathione S-transferase (GST), and monodehydroascorbate reductase (MDHAR) (Nadarajah, 2020). Collectively, investigating the association between environmental factors and plant antioxidant defense systems is of great significance. To date, existing studies have only documented flavonoid antioxidant activity in *E. wushanense*, whereas comprehensive evaluations on the antioxidant capacity of *Epimedium* species plants are scarce. Moreover, comparative antioxidant analyses among different tissues of *Epimedium* species have rarely been reported (Li et al., 2012).

Given these research gaps, the present study focused on three *Epimedium* species native to Shaanxi Province, China-*E. wushanense*, *E. brevicornu*, and *E. pubescens*-and conducted untargeted metabolomic profiling and antioxidant capacity assessments across root, stem, and leaf tissues. Meteorological data at the sampling site were simultaneously collected to characterize the local environmental background. Finally, the quality of Shaanxi-produced *Epimedium* was evaluated by comparing differential metabolites and antioxidant capacity among groups.

## 2 Materials and methods

### 2.1 Plant materials

Three-year-old plants of *E. wushanense*, *E. brevicornu* and *E. pubescens* were harvested in July 2025 from an *Epimedium* cultivation base in Jiuzhenba Village, Changling Town, Zhenba County, Hanzhong City, Shaanxi Province, China (32°27’15”N, 107°53’41”E). The samples were identified by Hong Zhang, professor at the Shaanxi Provincial Academy of Traditional Chinese Medicine. Root, stem, and leaf tissues were separately collected from each species. For every tissue, three biological replicates were performed. After rinsing with ultrapure water, all samples were immediately flash-frozen in liquid nitrogen and stored at −80 °C until metabolomic profiling and antioxidant activity assays.

### 2.2 Collection of meteorological data

Meteorological data for the sampling site, including atmospheric pressure, temperature, relative humidity, and precipitation, were obtained from the China Meteorological Data Network (https://data.cma.cn/), covering the period from July 2022 to July 2025. These data were used solely to characterize the local environmental background of the sampling site and were not intended to establish causal relationships between climatic factors and metabolite accumulation, given the single-time-point sampling design of this study.

### 2.3 Measurement of antioxidant activities

The contents of H_2_O_2_ (JL-T0898) and O_2_^-^ (JL-T0783), as well as the activities of SOD (JL-T0781), POD (JL-T0902) and CAT (JL-T0900) were determined using commercial kits purchased from Shanghai Jianglai Biotechnology Co., Ltd.

### 2.4 Metabolomics analysis

Plant tissues (~ 80 mg) were ground into a fine powder using a mortar and pestle after rapid freezing in liquid nitrogen. For metabolite extraction, 1000 μL of methanol/acetonitrile/H_2_O (2: 2: 1, v/v/v) was added to the homogenized solution. The mixture was centrifuged at 14, 000 g for 20 min at 4 °C. The supernatant was dried using a vacuum centrifuge and redissolved in 100 μL of acetonitrile/water (1: 1, v/v) for LC-MS analysis. The reconstituted samples were centrifuged at 14, 000 g for 15 min at 4 °C, and the supernatant was injected.

Analysis was performed using an Agilent 1290 Infinity UHPLC system coupled to an AB Sciex TripleTOF 6600 quadrupole time-of-flight mass spectrometer (Shanghai Applied Protein Technology Co., Ltd.) (Leibiger et al., 2025). Chromatographic separation was achieved on an ACQUITY UPLC BEH Amide column (2.1 mm × 100 mm, 1.7 µm, Waters, Ireland) using HILIC separation. The mobile phase consisted of A (25 mM ammonium acetate and 25 mM ammonium hydroxide in water) and B (acetonitrile). The gradient elution was programmed as follows: 95% B for 0.5 min, linearly reduced to 65% B in 4.5 min, reduced to 40% B in 0.5 min, maintained for 0.5 min, increased to 95% B in 0.5 min, and re-equilibrated for 2 min. ESI source conditions were set as follows: Ion Source Gas1 and Gas2 at 60 psi, curtain gas (CUR) at 30 psi, source temperature at 600 °C, and IonSpray Voltage Floating (ISVF) at ± 5500 V. MS data were acquired in both positive and negative modes. For MS-only acquisition, the m/z range was set to 60-1000 with an accumulation time of 0.20 s/spectrum. For auto MS/MS acquisition, the m/z range was 25-1000 with an accumulation time of 0.05 s/spectrum, using information-dependent acquisition (IDA) with high sensitivity mode (collision energy 35 V ± 15 eV).

To monitor analytical stability and repeatability, quality control (QC) samples were prepared by pooling 10 μL of supernatant from each sample extract. QC samples were injected after every five experimental samples throughout the analytical run. Raw MS data were converted to mzXML format using ProteoWizard MSConvert and processed using the open-source XCMS package (Yang C. et al., 2025) in R. Peak detection was performed using the centWave algorithm with the following parameters: m/z tolerance = 10 ppm, peakwidth = c (10, 60), and prefilter = c (10, 100). Peak grouping was conducted with bw = 5, mzwid = 0.025, and minfrac = 0.5. Isotope and adduct annotation were performed using the CAMERA package (Kuhl et al., 2011). Features with > 50% nonzero values in at least one group were retained for downstream analysis. Data were sum-normalized prior to multivariate statistical analysis.

Metabolite identification was performed following the Metabolomics Standards Initiative (MSI) reporting criteria (Fiehn et al., 2007), with four confidence levels: MSI 1 (confirmed by authentic standards), MSI 2 (putatively annotated by spectral matching or fragment interpretation), MSI 3 (assigned to compound class only), and MSI 4 (unannotated metabolites). A mass accuracy threshold of ≤ 5 ppm was applied for molecular formula assignment. Compounds with a mass error > 5 ppm or only one fragment ion were automatically downgraded to MSI 3; such metabolites were not excluded but assigned to the corresponding lower MSI identification level.

Chemical classification of the detected MS features was performed at MSI Level 3 using the ClassyFire tool (Djoumbou Feunang et al., 2016). Initially, exact masses and MS/MS fragmentation patterns were matched against public metabolite spectral libraries to obtain putative structural identifiers (InChIKeys). These identifiers were then input into ClassyFire to automatically assign features to specific chemical subclasses based on their structural properties. Features lacking reliable spectral matches were categorized as “unknown” or “undefined”, while those belonging to sparse, low-abundance families without a dominant group were aggregated into the “others” category. It is important to note that this classification provides putative family-level information rather than exact compound-level structural identification. Subsequently, MS features underwent manual curation based on characteristic MS/MS fragments and literature comparison to refine annotations to MSI Level 2 or 3, although no identities were validated using authentic reference standards. Finally, the annotated metabolites were further classified according to KEGG functional categories for pathway analysis.

### 2.5 Statistical analyses

Univariate statistical analyses were performed using GraphPad Prism 9.0. Mean differences between two independent groups were compared using Student’s t-test, and multiple-group comparisons were performed using one-way analysis of variance (ANOVA), followed by the least significant difference (LSD) *post-hoc* test for homogeneous variances or Dunnett’s T3 test for heterogeneous variances. Multivariate statistical analyses, including Pareto-scaled principal component analysis (PCA) and orthogonal partial least-squares discriminant analysis (OPLS-DA), were performed using the ropls package in R (Wang and Huang, 2023). Model robustness was evaluated by cross-validation and response permutation testing (n = 200). To address the sample size constraints, the number of cross-validation folds was automatically determined by the ropls package: it was set equal to the number of samples per group (i.e., 3-fold cross-validation for 3 vs. 3 comparisons). For each OPLS-DA model, the actual sample number (n = 3), number of cross-validation folds (n = 3), R^2^Y and Q^2^Y (**Supplementary Table 1**), are presented. To correct for multiple hypothesis testing across thousands of MS features, *p*-values were adjusted using the Benjamini-Hochberg false discovery rate (FDR) procedure. Differentially accumulated metabolites (DAMs) were primarily defined by FDR < 0.05 and fold change (FC > 1.2 or < 0.833); variable importance in projection (VIP > 1) derived from the OPLS-DA model was retained as a supplementary criterion to support feature prioritization. Pearson correlation analysis was performed using the corrplot package in R to assess pairwise correlations between antioxidant enzyme activities, reactive oxygen species and key secondary metabolite abundances. Pairwise *p*-values were adjusted using the Benjamini-Hochberg FDR correction, and statistical significance was defined as adjusted *p* < 0.05.

## 3 Results

### 3.1 Meteorological factors

Meteorological data for the *Epimedium* sampling site from July 2022 to July 2025 are summarized in **Figure 1**, providing environmental context for interpreting the metabolomic and antioxidant profiles of the three species. As shown in **Figure 1A**, atmospheric pressure remained relatively stable over the monitoring period, with mean values ranging from 955 to 958 hPa, maximum values ranging from 966 to 969 hPa, and minimum values ranging from 942 to 947 hPa. As shown in **Figure 1B**, temperature exhibited clear seasonal variation, with mean values ranging from 16 °C to 19 °C, mean maximum temperatures ranging from 21 °C to 23 °C, and mean minimum temperatures ranging from 12 °C to 16 °C. Notably, all temperature indicators reached their lowest levels in 2023. As shown in **Figure 1C**, relative humidity ranged between 47.9% and 91.1% throughout the monitoring period. As shown in **Figure 1D**, precipitation ranging from 0.1 to 188.2 mm, with the highest rainfall consistently occurring from July to September each year. Conversely, negligible precipitation was recorded in January 2023 and December 2024.

**Figure 1:**
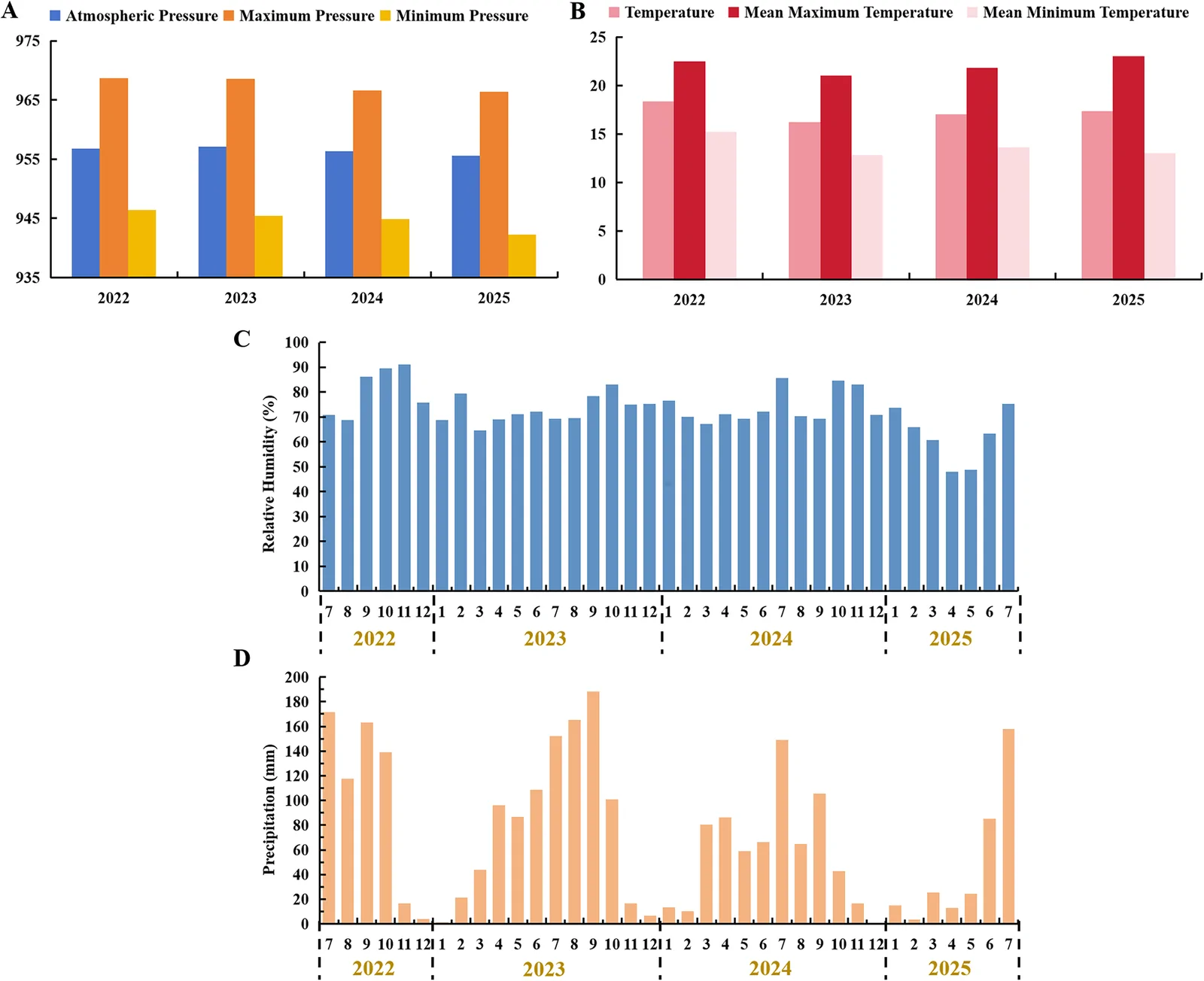
Dynamic meteorological factors in Zhenba County, Hanzhong City, Shaanxi Province, China from July 2022 to July 2025. **(A)** Atmospheric pressure, maximum pressure and minimum pressure (hPa). **(B)** Temperature, mean maximum temperature and mean minimum temperature (°C). **(C)** Relative humidity. **(D)** Precipitation.

### 3.2 Antioxidant activities of Epimedium species

The ROS contents and antioxidant enzyme activities of the three *Epimedium* species are summarized (**Figure 2**). For H_2_O_2_, root tissue consistently exhibited the highest content within each species, followed by leaf and then stem (**Figure 2A**). Across species, *E. brevicornu* showed the highest H_2_O_2_ content in each corresponding tissue, followed by *E. pubescens* and *E. wushanense*. The O_2_^-^ content followed a similar tissue-specific pattern, with roots showing the highest levels within each species (**Figure 2B**). However, the species-specific pattern of O_2_^-^ content varied by tissue: in roots, *E. pubescens* had the highest O_2_^-^ content, followed by *E. wushanense* and *E. brevicornu*; in stems, *E. brevicornu* ranked highest, followed by *E. pubescens* and *E. wushanense*; in leaves, *E. pubescens* showed the highest content, followed by *E. brevicornu* and *E. wushanense*.

**Figure 2:**
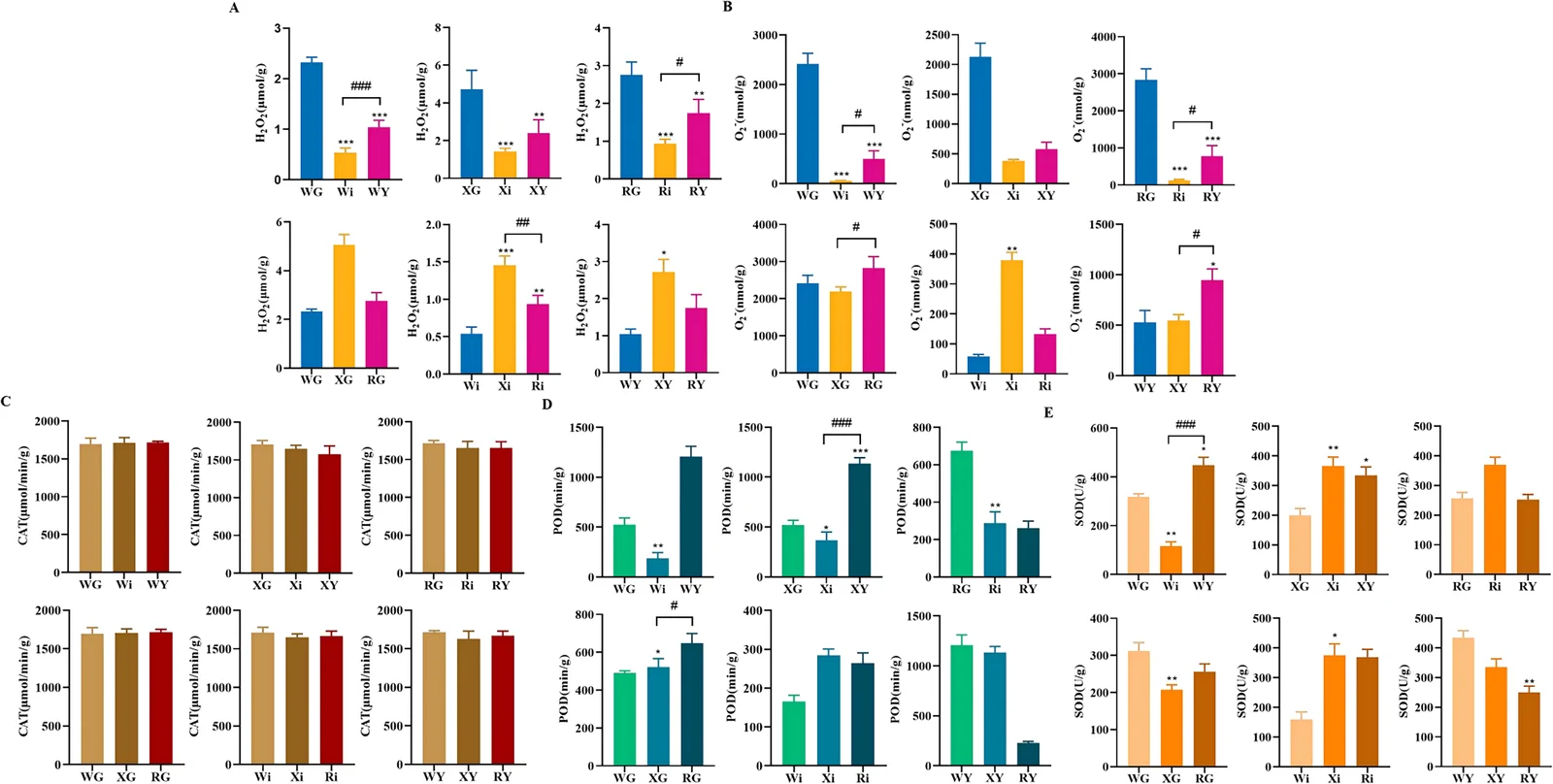
Antioxidant capacity of three Epimedium species plant. **(A)** H_2_O_2_ content. **(B)** O_2_- content. **(C)** CAT activity. **(D)** POD activity. **(E)** SOD activity. WG: The root of *Epimedium wushanense*. Wi: The stem of *E. wushanense*. WY: The leaf of *E. wushanense*. XG: The root of *Epimedium brevicornu*. Xi: The stem of *E. revicornu*. XY: The leaf of *E. brevicornu*. RG: The root of *Epimedium pubescens*. Ri: The stem of *E. pubescens*. RY: The leaf of E. pubescens. The same for the following figures. Compared with WG, the “*” stands for the significant difference at *p*<0.05; the “**” stands for the significant difference at *p*<0.01; the “***” stands for the significant difference at *p*<0.001. The “#” stands for the significant difference at *p*<0.05; the “##” stands for the significant difference at *p*<0.01; the “###” stands for the significant difference at *p*<0.001.

No significant differences in CAT activity were observed either across tissues within each species or across species within each tissue (**Figure 2C**). For POD activity, *E. wushanense* and *E. brevicornu* showed the highest activity in leaves, followed by roots and then stems, whereas *E. pubescens* exhibited the highest activity in roots, followed by stems and leaves (**Figure 2D**). Across species, the highest POD activity was detected in the roots of *E. pubescens*, the stems of *E. brevicornu*, and the leaves of *E. wushanense*. For SOD activity, tissue-specific patterns also differed among species: *E. wushanense* showed the highest activity in leaves, whereas both *E. brevicornu* and *E. pubescens* exhibited the highest activity in stems (**Figure 2E**). Across species, *E. wushanense* had the highest SOD activity in both roots and leaves compared with the other two species; in stems, the activities of *E. brevicornu* and *E. pubescens* were comparable and both significantly higher than that of *E. wushanense*.

### 3.3 Metabolite profiling of Epimedium species

Metabolite variations were compared across two dimensions: between different tissues within each *Epimedium* species and among the three *Epimedium* species within the same tissue. A total of 2730 MS features were detected, of which 2268 received chemical-class assignments (MSI Level 3) and were grouped into multiple chemical categories; 46 features were further putatively annotated at the compound level (MSI Level 2 or 3) via MS/MS fragmentation and literature comparison (**Figure 3**).

**Figure 3:**
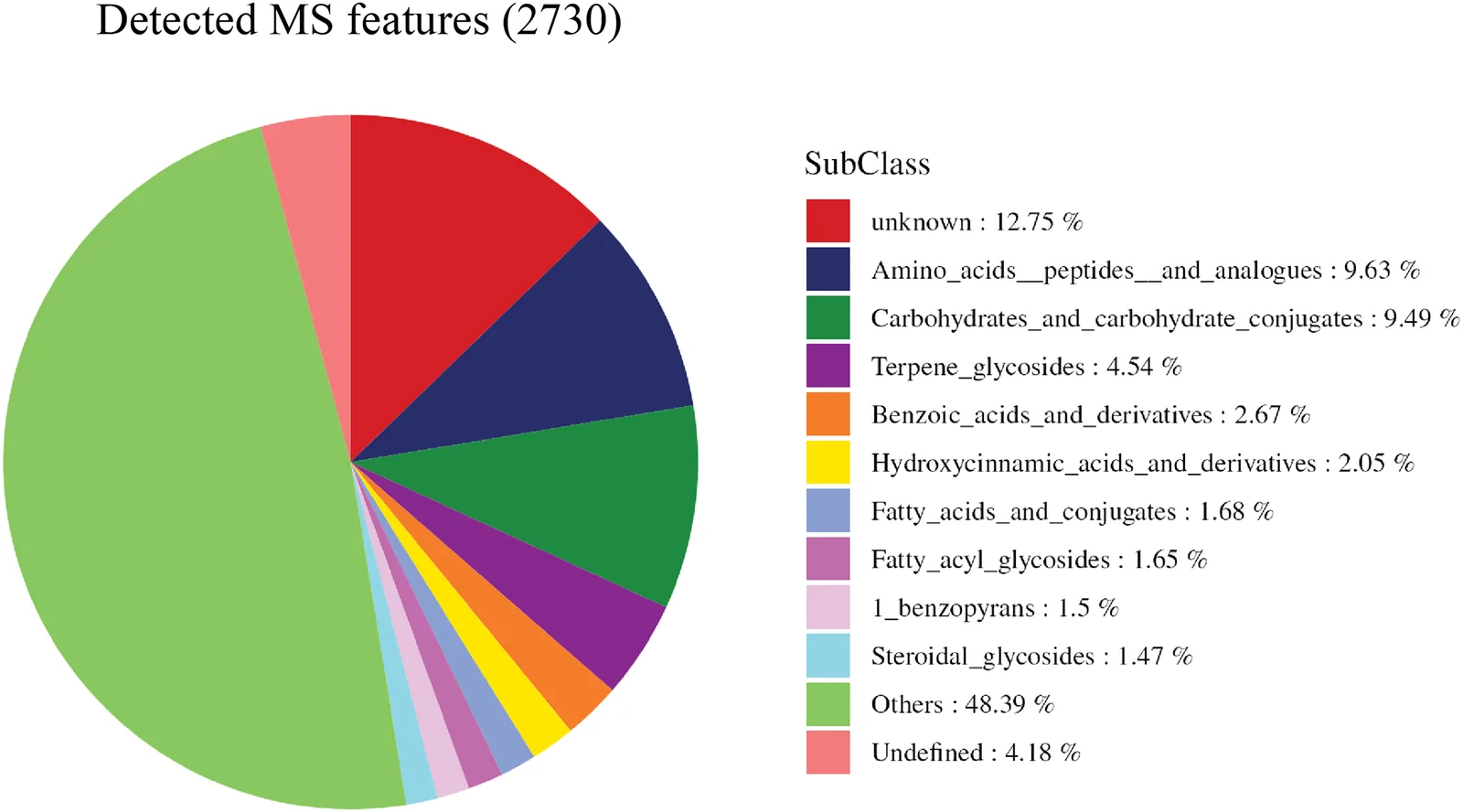
Total detected metabolites of *Epimedium* species plant.

To further validate interspecies differences and identify metabolites contributing to group separation, pairwise orthogonal partial least-squares discriminant analysis (OPLS-DA) was performed across all tissue types and species pairs among the three *Epimedium* species. Orthogonal partial least-squares discriminant analysis (OPLS-DA) score plot are presented in **Supplementary Figures 1**, **2** and **3**, clear separation was observed for each species pair in both positive and negative ionization modes. All OPLS-DA models demonstrated good goodness of fit and predictive performance (R²Y > 0.9, Q²Y > 0.8) (**Supplementary Table 1**).

As shown in the PCA score plots, samples were generally separated into three distinct groups corresponding to the comparison groups in each plot (**Supplementary Figure 4**). PC1 accounted for 23.7% to 51% of the total variance across all comparison groups. Most sample groups exhibited clear separation, with the exception of partial overlap between *E. wushanense* and *E. pubescens* in the positive ionization mode for stem tissue, suggesting similar metabolite compositions in the stems of these two species.

### 3.4 Analysis of differential metabolites in Epimedium species

Metabolite pairs with a correlation coefficient |*r*| > 0.8 and FDR adjust-*p* < 0.05 were selected to construct the chord diagrams of correlations among DAMs (**Figure 4**). In *E. pubescens*, 58 compounds (negative mode) and 32 compounds (positive mode) exhibited potential associations, with the top three being plantamajoside, chlorogenic acid and citraconic acid (negative mode), and cellopentaose, ginkgolide c and reticuline (positive mode) (**Figure 4A**). In *E. wushanense*, 48 compounds (negative mode) and 38 compounds (positive mode) exhibited potential associations, with the top three being 2-(4-Hydroxyphenyl)ethyl(2*S*, 3*Z*, 4*S*)-4-(2-(2-(3, 4-dihydroxyphenyl)ethoxy)-2-oxoethyl)-2-(*β*-*D*-glucopyranosyloxy)-3-(2-hydroxyethylidene)-3, 4-dihydro-2*H*-pyran-5-carboxylate, chromomoric acid b and 3, 4-dihydroxy-5-[(2*S*, 3*R*, 4*S*, 5*S*, 6*R*)-3, 4, 5-trihydroxy-6-[[4-(2-hydroxypropan-2-*yl*)cyclohexene-1-carbonyl]oxymethyl]oxan-2-yl]oxybenzoic acid (negative mode), and carboline metabolite, kotanin and schizandrin b (positive mode) (**Figure 4B**). In *E. brevicornu*, 53 compounds (negative mode) and 44 compounds (positive mode) exhibited potential associations, with the top three being sucrose, chlorogenic acid and 3*a*-galactobiose-CH_3_ (negative mode), and *B*-pentasaccharide, trilinolein and schizandrin b (positive mode) (**Figure 4C**). In roots, 12 compounds (negative mode) and 21 compounds (positive mode) showed potential associations, with the top three being melibiose, sucrose and syringaresinol (negative mode), and reticuline, acanthoside b and trilinolein (positive mode) (**Figure 4D**). In stems, 59 compounds (negative mode) and 30 compounds (positive mode) showed potential associations, with the top three being picraquassioside a, (2*S*, 3*R*)-2, 3, 4-trihydroxybutyl 2, 6-*di*-*O*-acetyl-3 -*O*-dodecanoyl-*β*-*D*-mannopyranoside and *D*-saccharic acid 1, 4-lactone (negative mode), and trigonelline, paeonolide and (*s*)-salsoline (positive mode) (**Figure 4E**). In leaves, 35 compounds (negative mode) and 34 compounds (positive mode) showed potential associations, with the top three being *D*-saccharic acid 1, 4-lactone, osmanthuside h and 3’, 5’-*di*-*β*-*D*-glucopyranosylphloretin (negative mode), and reticuline, trans-ferulic acid and (1*R*)-chrysanthemolactone (positive mode) (**Figure 4F**).

**Figure 4:**
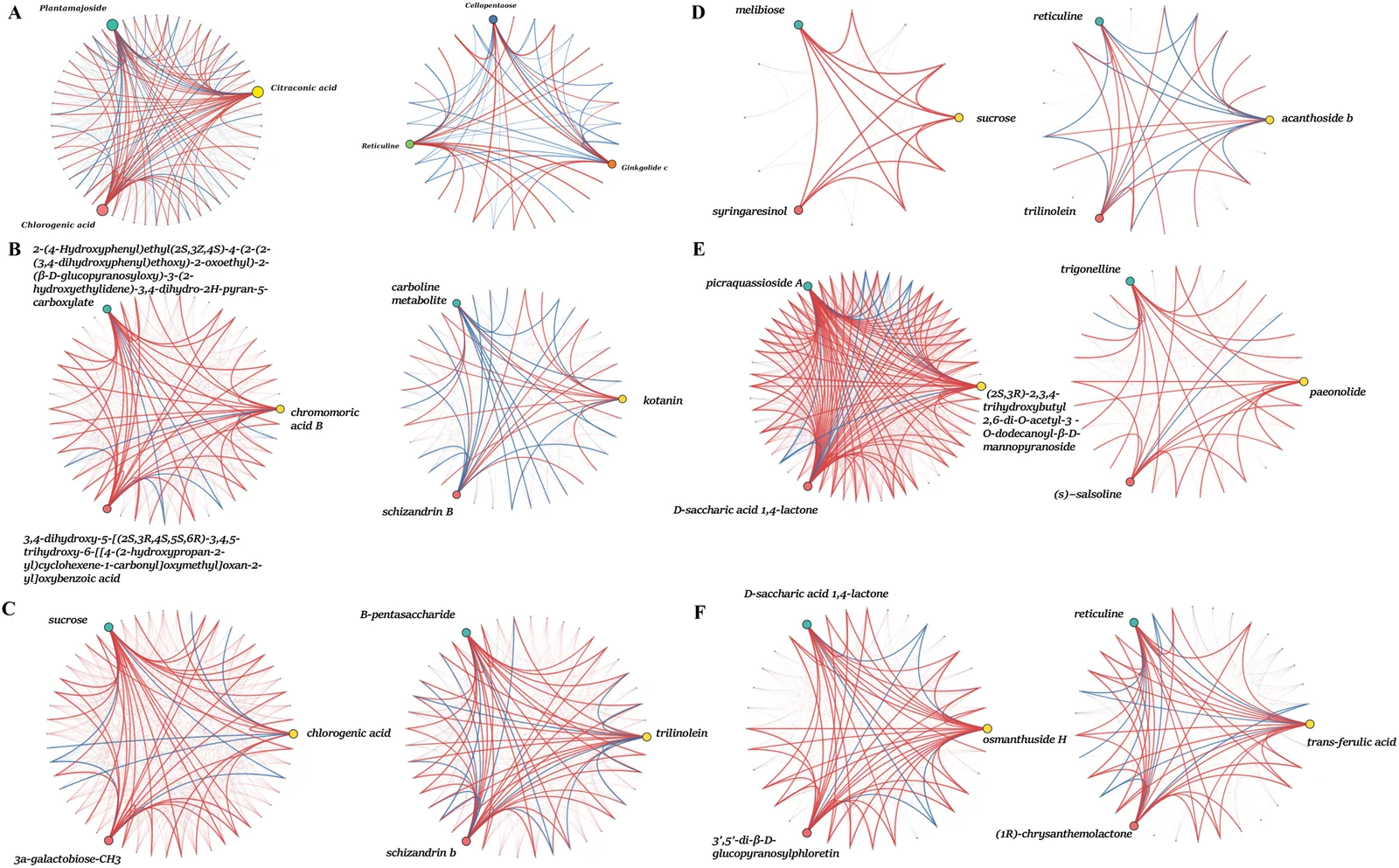
Correlation of DAMs: **(A)** Correlation of DAMs among roots, stems, and leaves of *E. pubescens*. **(B)** Correlation of DAMs among roots, stems, and leaves of *E. wushanense*. **(C)** Correlation of DAMs among roots, stems, and leaves of *E. brevicornu*. **(D)** Correlation of DAMs in roots of *E. wushanense*, *E. brevicornu*, and *E. pubescens*. **(E)** Correlation of DAMs in stems of *E. wushanense*, *E. brevicornu*, and *E. pubescens*. **(F) ** Correlation of DAMs in leaves of *E. wushanense*, *E. brevicornu*, and *E. pubescens*. The negative ion mode (left) and positive ion mode (right).

After merging the DAMs screened in positive and negative ion modes, KEGG pathway annotation and analysis among different groups were performed (**Figure 5**) (Zhong et al., 2026). For *E. pubescens*, the top three pathways were citric acid cycle (TCA cycle) stilbenoid, diarylheptanoid and gingerol biosynthesis, and other carbon fixation pathways, with key metabolites including malate, citrate, cis-aconitate, chlorogenic acid, curcumin and 3-*O*-*p*-coumaroylquinic acid. For *E. wushanense*, the top three pathways were fatty acid biosynthesis, biosynthesis of unsaturated fatty acids and riboflavin metabolism, with key metabolites including palmitic acid, oleic acid, myristic acid, arachidic acid, flavin mononucleotide (FMN) and (-)-riboflavin. For *E. brevicornu*, the top three pathways were galactose metabolism, stilbenoid, diarylheptanoid and gingerol biosynthesis, and ABC transporters, with key metabolites including sucrose, myo-inositol, galactinol, melibiose, chlorogenic acid, curcumin, 3-*O*-*p*-coumaroylquinic acid, (-)-riboflavin and maltotriose.

**Figure 5:**
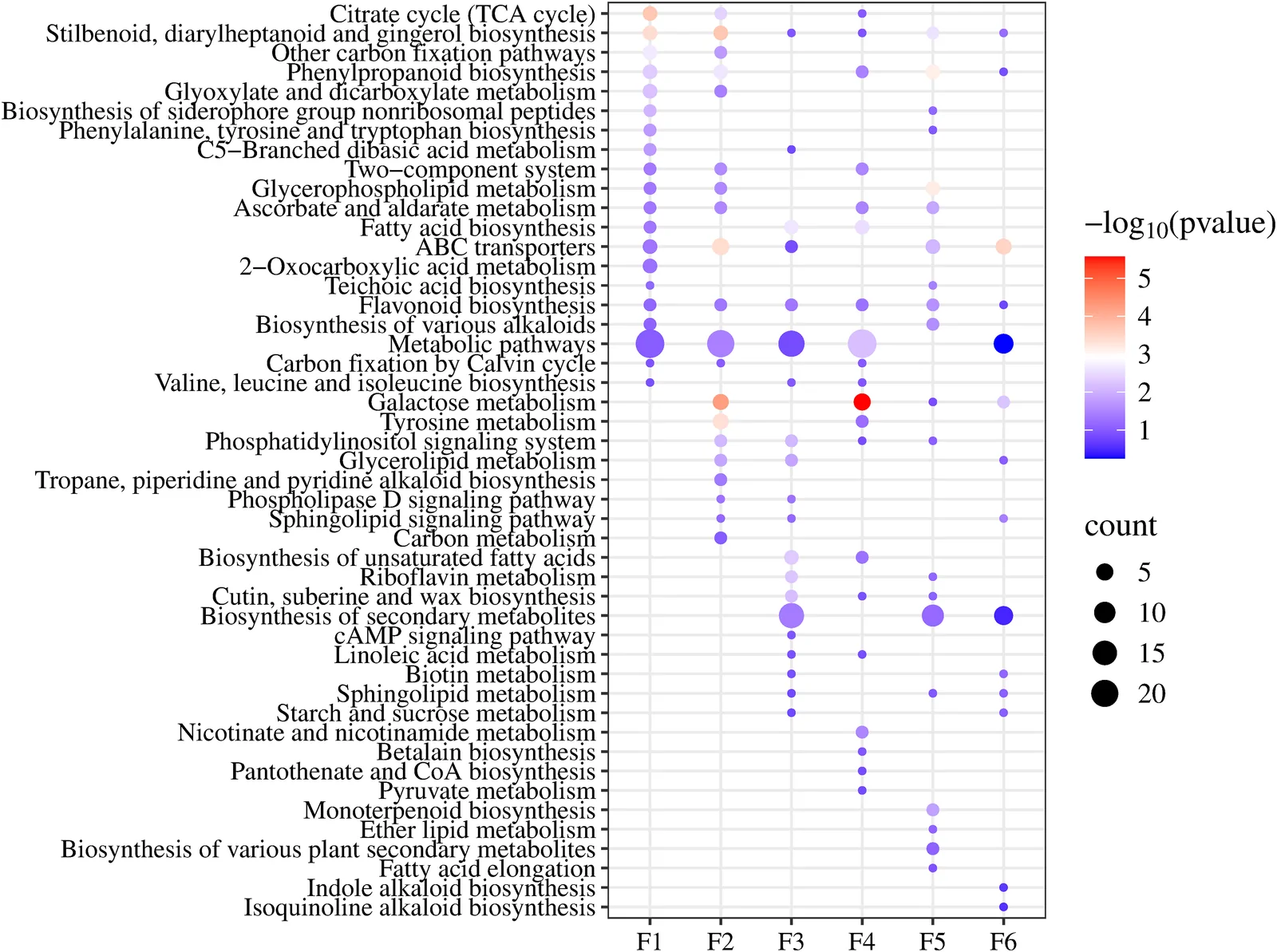
KEGG enrichment analysis of DAMs (TOP 20). (F1) KEGG enrichment analysis of DAMs among roots, stems, and leaves of *E. pubescens*. (F2) KEGG enrichment analysis of DAMs among roots, stems, and leaves of *E. brevicornu*. (F3) KEGG enrichment analysis of DAMs among roots, stems, and leaves of *E. wushanense*. (F4) KEGG enrichment analysisn of DAMs in stems of *E. wushanense*, *E. brevicornu*, and *E. pubescens*. (F5) KEGG enrichment analysis of DAMs in leaves of *E. wushanense*, *E. brevicornu*, and *E. pubescens*. (F6) KEGG enrichment analysis of DAMs in roots of *E. wushanense*, *E. brevicornu*, and *E. pubescens*.

For root tissue across the three species, the top three enriched pathways were ABC transporters, galactose metabolism and stilbenoid, diarylheptanoid and gingerol biosynthesis, with key metabolites including sucrose, 1, 4-*D*-xylobiose, maltotriose, melibiose and chlorogenic acid. For stem tissue across the three species, the top three enriched pathways were galactose metabolism, fatty acid biosynthesis and biosynthesis of various plant secondary metabolites, with key metabolites including myo-inositol, raffinose, galactonic acid, galactinol, stachyose, palmitic acid, dodecanoic acid, and myristic acid. For leaf tissue across the three species, the top three enriched pathways were glycerophospholipid metabolism, phenylpropanoid biosynthesis and stilbenoid, diarylheptanoid and gingerol biosynthesis, with key metabolites including choline cation, glycerophosphocholine, 1-Palmitoyl-sn-glycero-3-phosphocholine, chlorogenic acid, trans-Ferulic acid, and 3-*O*-*p*-coumaroylquinic acid.

### 3.5 Metabolite set enrichment analysis

All metabolites in each comparison group were subjected to MSEA (Pei et al., 2025), which enables the detection of pathway-level enrichment even when individual metabolites show modest abundance changes (**Figure 6**). For *E. pubescens*, three enriched metabolite sets-pentose and glucuronate interconversions, glutathione metabolism, and biosynthesis of cofactors-warrant attention, as they may be related to the biosynthesis of bioactive components in this species. For *E. wushanense*, nicotinate and nicotinamide metabolism, glycerolipid metabolism, and biosynthesis of nucleotide sugars were enriched, which may be involved in bioactive component biosynthesis. For *E. brevicornu*, glyoxylate and dicarboxylate metabolism, benzenoid biosynthesis, and plant hormone signal transduction were identified as enriched sets, potentially related to active ingredient biosynthesis.

**Figure 6:**
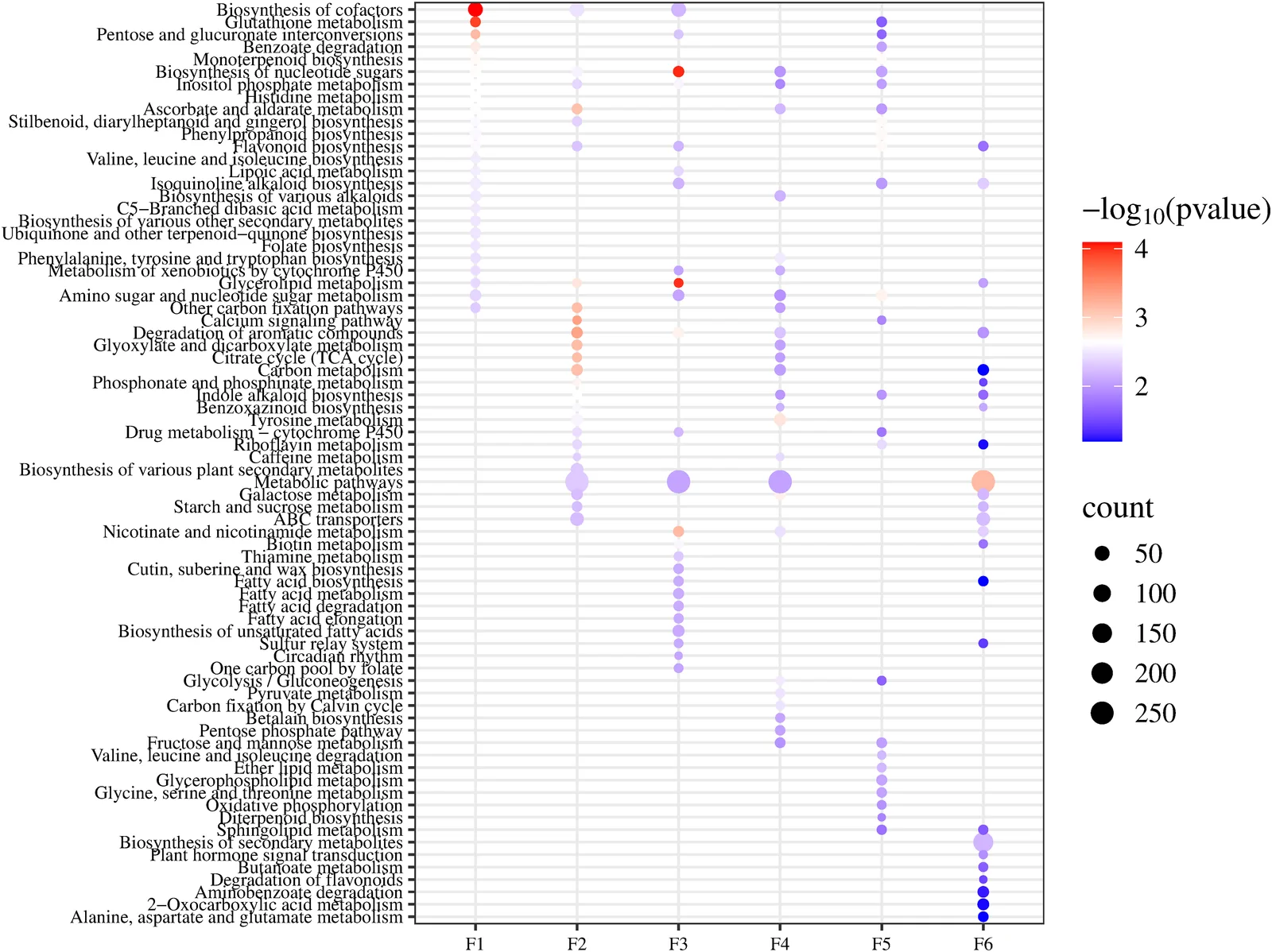
Metabolite set enrichment analysis (MSEA) (TOP 25). (F1) KEGG enrichment analysis of DAMs among roots, stems, and leaves of *E. pubescens*. (F2) KEGG enrichment analysis of DAMs among roots, stems, and leaves of *E. brevicornu*. (F3) KEGG enrichment analysis of DAMs among roots, stems, and leaves of *E. wushanense*. (F4) KEGG enrichment analysisn of DAMs in stems of *E. wushanense*, *E. brevicornu*, and *E. pubescens*. (F5) KEGG enrichment analysis of DAMs in leaves of *E. wushanense*, *E. brevicornu*, and *E. pubescens*. (F6) KEGG enrichment analysis of DAMs in roots of *E. wushanense*, *E. brevicornu*, and *E. pubescens*.

For root tissue across the three species, ABC transporters, 2-oxocarboxylic acid metabolism, and metabolic pathways were the most prominently enriched sets, which may be relevant to secondary metabolism in *Epimedium* species. For stem tissue, glycolysis/gluconeogenesis, galactose metabolism, and tyrosine metabolism were enriched, potentially providing precursors for *Epimedium* secondary metabolism. For leaf tissue, flavonoid biosynthesis, stilbenoid/diarylheptanoid/gingerol biosynthesis, and amino sugar/nucleotide sugar metabolism were enriched, which may be relevant to the biosynthesis of characteristic secondary metabolites.

### 3.6 Analysis of key secondary metabolic pathways in Epimedium species

The abundance variations of compounds involved in key secondary metabolic pathways of *Epimedium* species are illustrated in **Figure 7**. Five pathways were examined: phenylpropanoid biosynthesis (map00940), flavonoid biosynthesis (map00941), anthocyanin biosynthesis (map00942), isoflavonoid biosynthesis (map00943), and flavone and flavonol biosynthesis (map00944). A total of 18 metabolites associated with these pathways were detected, including 11 in the negative ion mode and 7 in the positive ion mode.

**Figure 7:**
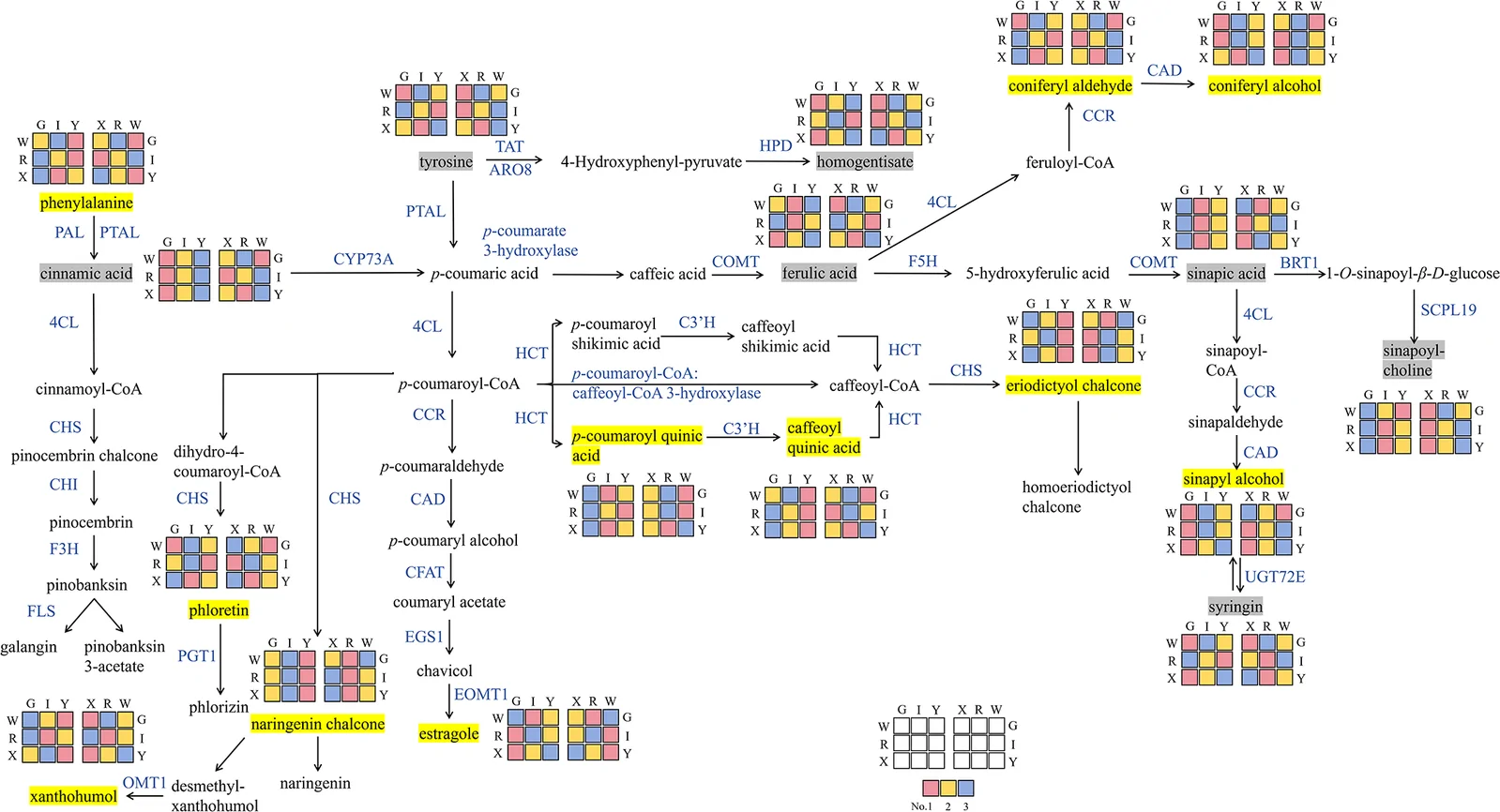
Analysis of key regulatory pathways of flavonoid biosynthesis. Metabolites highlighted in yellow were identified in negative ion mode, whereas those in gray were identified in positive ion mode. The left panel (3x3 grid) illustrates the metabolite contents in different parts of the three *Epimedium* species, while the right panel shows the contents in the same part across the three species. Pink indicates the highest content, followed by yellow, and blue represents the lowest. G: the part of root. i: the part of stem. Y: the part of leaf. X: *Epimedium brevicornu*. R: *Epimedium pubescens*. W: *Epimedium wushanense*.

For *E. wushanense*, 6 metabolites-phenylalanine, xanthohumol, naringenin chalcone, caffeoylquinic acid, eriodictyol chalcone, and sinapoylcholine-were most abundant in leaves; 8 metabolites-cinnamic acid, phloretin, tyrosine, homogentisate, coniferyl aldehyde, coniferyl alcohol, sinapyl alcohol, and syringin-were most abundant in roots; and the remaining 4 (estragole, ferulic acid, sinapic acid, and *p*-coumaroylquinic acid) were most abundant in stems. For *E. pubescens*, 10 metabolites were most abundant in leaves (phenylalanine, phloretin, naringenin chalcone, tyrosine, *p*-coumaroylquinic acid, caffeoylquinic acid, homogentisate, coniferyl aldehyde, eriodictyol chalcone, and syringin), 4 in roots (cinnamic acid, estragole, coniferyl alcohol, and sinapyl alcohol), and four in stems (xanthohumol, ferulic acid, sinapic acid, and sinapoylcholine). For *E. brevicornu*, 8 metabolites were most abundant in stems (phenylalanine, phloretin, tyrosine, sinapic acid, *p*-coumaroylquinic acid, coniferyl aldehyde, sinapoylcholine, and coniferyl alcohol), 6 in roots (cinnamic acid, estragole, homogentisate, syringin, sinapyl alcohol, and ferulic acid), and 4 in leaves (xanthohumol, naringenin chalcone, caffeoylquinic acid, and eriodictyol chalcone). Notably, cinnamic acid, naringenin chalcone, and sinapic acid showed consistent tissue-specific abundance trends across all three species.

In roots, phenylalanine, cinnamic acid, phloretin, *p*-coumaroylquinic acid, caffeoylquinic acid, coniferyl aldehyde, coniferyl alcohol, and sinapyl alcohol were significantly more abundant in *E. wushanense* than in the other two species. In stems, 12 metabolites-phenylalanine, phloretin, tyrosine, sinapic acid, coniferyl aldehyde, sinapoylcholine, coniferyl alcohol, cinnamic acid, homogentisate, sinapyl alcohol, caffeoylquinic acid, and eriodictyol chalcone-were most abundant in *E. brevicornu*. In leaves, phloretin, tyrosine, *p*-coumaroylquinic acid, coniferyl aldehyde, cinnamic acid, homogentisate, syringin, ferulic acid, naringenin chalcone, and caffeoylquinic acid were significantly more abundant in *E. pubescens* than in the other two species.

### 3.7 Analysis of key metabolisms in Epimedium species

Furthermore, 46 detected MS features were putatively annotated at the compound level (MSI Level 2 or 3) using MS/MS fragmentation patterns and literature comparison. Among which 42 were flavonoids, 2 were phenolic acids, 1 was a phenylpropanoid and 1 was an alkaloid, information on the retention time, molecular formula, secondary mass spectral fragment ions and compound name of these chemical constituents is presented in **Table 1**. Flavonoids, the most abundant constituents in *Epimedium* species plant, mainly included flavonol glycosides and free flavonols. The 2 annotated phenolic acids were chlorogenic acid and 5-*p*-coumaroyl quinic acid, and the single annotated alkaloid was magnoflorine.

**Table 1:** Putative compound-level annotations of 46 detected MS features in *Epimedium* L. plants.

No.	t_R_(min)	Formula	Precursor ions	Calc. mass	Mass	Error (δ/ppm)	Fragment ions	Identification	Types	MSI
1	2.42	C_33_H_40_O_16_	[M-H]^-^	691.2204	691.2272	9.8	383.1132; 368.0895; 325.0348; 313.0357; 175.0029; 545.1645; 529.1714	Sagittasine c	Flavonoids	Level 3
2	2.54	C_39_H_48_O_19_	[M-H]^-^	819.2679	819.2738	7.2	367.1189; 513.1770	3’’’-carbonyl-2’’-*β*-*L*-quinovosyl-icariin	Flavonoids	Level 3
3	0.78	C_39_H_50_O_20_	[M-H]^-^	837.2788	837.2841	6.3	61.9872; 675.2335	Acuminatoside	Flavonoids	Level 3
4	2.24	C_33_H_40_O_15_	[M-H]^-^	675.2276	675.2297	3.1	367.1181; 657.2178	Baohuoside VII	Flavonoids	Level 2
5	1.26	C_33_H_40_O_15_	[M-H]^-^	675.2270	675.2282	1.8	367.1138	Icariin	Flavonoids	Level 3
6	5.45	C_32_H_38_O_15_	[M-H]^-^	661.211	661.2118	1.2	353.1039	Epimedoside a	Flavonoids	Level 3
7	3.11	C_38_H_48_O_20_	[M-H]^-^	823.2632	823.2669	4.5	661.2113; 703.2072	Rouhuoside	Flavonoids	Level 2
8	1.82	C_21_H_20_O_10_	[M-H]^-^	431.0975	431.0971	-0.9	284.0329; 255.0295	Kaempferol-3- -rhamnoside	Flavonoids	Level 2
9	2.89	C_32_H_38_O_16_	[M-H]^-^	677.2056	677.2075	2.8	515.1566; 353.1028	Grandifloroside b	Flavonoids	Level 2
10	2.36	C_27_H_30_O_14_	[M-H]^-^	577.1546	577.1571	4.3	431.0947	Kaempferitrin	Flavonoids	Level 3
11	5.89	C_21_H_20_O_11_	[M-H]^-^	447.0922	447.0923	0.2	301.0351; 285.0402; 284.0344	Astragalin	Flavonoids	Level 2
12	5.85	C_21_H_20_O_12_	[M-H]^-^	463.0873	463.0871	-0.4	271.0248; 301.0348	Hyperoside	Flavonoids	Level 2
13	3.92	C_26_H_36_O_12_	[M-H]^-^	539.2116	539.2121	0.9	165.0543; 195.0662; 343.1392; 491.1851	Ikarisoside E6	Flavonoids	Level 2
14	2.63	C_16_H_18_O_9_	[M-H]^-^	353.0844	353.0883	11.0	135.0437; 179.0492; 191.0542	Chlorogenic acid	Phenolic acids	Level 3
15	3.66	C_16_H_18_O_8_	[M-H]^-^	337.0909	337.0923	4.2	137.0255; 163.0374; 173.0479	5-*p*-Coumaroyl quinic acid	Phenolic acids	Level 2
16	2.75	C_20_H_23_NO_4_	[M-H]^-^	340.1535	340.1544	2.6	340.1513	Magnoflorine	Alkaloids	Level 3
17	3.51	C_33_H_40_O_19_	[M-H]^-^	739.2075	739.2085	1.4	593.1518	Kaempferol-3-*O*-rhamnopyranosyl-glucopyranosyl-7-*O*-rhamnopyranoside	Flavonoids	Level 3
18	2.29	C_26_H_36_O_11_	[M-H]^-^	523.2180	523.2181	0.2	331.1549; 361.1640	Icariside E3	Flavonoids	Level 2
19	2.36	C_27_H_30_O_14_	[M-H]^-^	577.1517	577.1563	8.0	431.0980; 285.0410	Kaempferol-3,7-O-rhamnoside	Flavonoids	Level 3
20	5.33	C_21_H_20_O_11_	[M-H]^-^	447.0908	447.0927	4.2	300.0233; 301.0395	Quercetin-3-*O*-*α*-*L*-rhamnoside	Flavonoids	Level 2
21	3.29	C_40_H_50_O_20_	[M-H]^-^	849.2810	849.2848	4.5	645.2160	Demethylanhydroicaritin-3-*O*-*di*-rhamnopyranosyl-7-*O*-(acetyl) glucopyranoside	Flavonoids	Level 3
22	2.65	C_39_H_48_O_20_	[M-H]^-^	835.2630	835.2689	7.1	673.2118	4’,5-dihydroxyl-8-(3,3-dimethylallyl)-flavonol-3-*O*-[*β*-*D*-xylopyranosyl(1→3)-4-*O*-acetyl-*α*-*L*-rhamnopyranoside]-7-*O*-*β*-*D*-glucopyranoside	Flavonoids	Level 3
23	3.03	C_39_H_50_O_20_	[M-H]^-^	837.2795	837.2798	0.4	367.1123; 675.2223	Isomer of Epimedin a	Flavonoids	Level 2
24	1.64	C_39_H_46_O_18_	[M-H]^-^	801.2594	801.2572	-2.7	367.1195; 513.1739; 639.2085	Anhydroicaritin-3-*O*-(3acetyl) rhamnopyranosyl-7-*O*-glucopyranoside	Flavonoids	Level 2
25	1.58	C_26_H_28_O_10_	[M-H]^-^	499.1599	499.1573	-5.2	353.1027	Ikarisoside a	Flavonoids	Level 3
26	1.6	C_27_H_30_O_11_	[M-H]^-^	529.1685	529.1690	0.9	383.1157	Caohuoside c	Flavonoids	Level 3
27	1.02	C_33_H_40_O_15_	[M-H]^-^	675.2284	675.2281	-0.4	367.1191; 513.1747	Sagittatoside a	Flavonoids	Level 2
28	1.05	C_27_H_30_O_11_	[M-H]^-^	529.1714	529.1709	-0.9	367.1183	Icariside I	Flavonoids	Level 3
29	2.31	C_32_H_38_O_14_	[M-H]^-^	645.2173	645.2192	2.9	352.0948	Sagittatoside b	Flavonoids	Level 3
30	3.38	C_35_H_42_O_16_	[M-H]^-^	717.2359	717.2395	5.0	352.0926; 367.1185; 513.1772	Anhydroicaritin-3-*O*-rhamnopyranosyl-(acetyl) glucopyranoside	Flavonoids	Level 2
31	2.05	C_27_H_30_O_10_	[M-H]^-^	513.1759	513.1741	-3.5	323.0924; 351.0874; 366.1120	Icariside II	Flavonoids	Level 2
32	0.43	C_20_H_18_O_6_	[M-H]^-^	353.1011	353.1022	3.1	353.1018	Demethylicaritiin	Flavonoids	Level 3
33	2.27	C_21_H_20_O_6_	[M-H]^-^	367.1180	367.1186	1.6	309.0401; 352.261	Icaritin	Flavonoids	Level 2
34	1.94	C_26_H_28_O_11_	[M+H]^+^	517.1704	517.1706	0.4	299.0551; 355.1183	Epimedium glycoside c	Flavonoids	Level 2
35	0.82	C_8_H_8_O_3_	[M+H]^+^	151.0398	151.03984	0.3	108.0207	Isovanillin	benzenoid	Level 3
36	3.47	C_33_H_40_O_16_	[M+H]^+^	693.2388	693.2391	0.4	385.1257;	Sagittasine c	Flavonoids	Level 3
37	3.14	C_39_H_50_O_20_	[M+H]^+^	839.2997	839.2945	-6.2	367.1177; 531.1817	Acuminatoside	Flavonoids	Level 3
38	2.75	C_26_H_28_O_14_	[M+H]^+^	565.1566	565.1531	-6.2	287.0581	Kaempferol-7-*O*-rhamnopyranosyl-xylopyranoside	Flavonoids	Level 3
39	3.16	C_38_H_48_O_19_	[M+H]^+^	809.2852	809.2868	2.0	355.1167; 517.1691; 663.2255	Diphylloside b	Flavonoids	Level 2
40	3.16	C_32_H_38_O_15_	[M+H]^+^	663.2268	663.2268	0.0	355.1180; 517.1710	Epimedoside	Flavonoids	Level 2
41	3.14	C_39_H_50_O_20_	[M+H]^+^	839.2960	839.2945	-1.8	385.1266; 547.1817	Sagittasine b or its isomers	Flavonoids	Level 2
42	2.57	C_33_H_40_O_16_	[M+H]^+^	693.2388	693.2392	0.6	369.1337; 367.1165; 531.1862	Caohuoside	Flavonoids	Level 2
43	2.84	C_38_H_48_O_19_	[M+H]^+^	809.2888	809.2853	-4.3	369.1336; 531.1880; 677.2455	Epimedin b	Flavonoids	Level 2
44	3.46	C_39_H_50_O_19_	[M+H]^+^	823.3049	823.3021	-3.4	313.0722; 369.1347; 531.1875; 677.2475	Epimedin c	Flavonoids	Level 2
45	1.98	C_39_H_46_O_18_	[M+H]^+^	803.2729	803.2761	4.0	313.0732; 369.1343; 531.1868	Anhydroicaritin-3-*O*-(3acetyl)rhamnopyranosyl-7-*O*-glucopyranoside	Flavonoids	Level 2
46	3.67	C_32_H_38_O_15_	[M+H]^+^	663.2268	663.2263	-0.8	355.1176	Ikarisoside b	Flavonoids	Level 3

*Rows 1–33: precursor ion recorded as [M−H]⁻ (negative ionization mode); Rows 34–46: precursor ion recorded as [M+H]⁺ (positive ionization mode).

In this study, sagittasine c and chlorogenic acid were selected as representative flavonoid and phenolic acid compounds, respectively, and their mass spectrometric fragmentation pathways are illustrated (**Figure 8**). Compound 1 (t_R_ = 2.42 min) exhibited quasi-molecular ions at *m/z* 691.2204 [M-H]^-^ in the mass spectrum. The fragment ion at *m/z* 545.16455 was presumably generated by the loss of one rhamnose moiety at the C-3 position; the fragment at *m/z* 529.17285 was attributed to the elimination of one glucose moiety at the C-7 position, corresponding to [M-Glc-H]^-^. Simultaneous loss of one Glc and one Rha moiety gave rise to the fragment ion at *m/z* 383.11346 [M-Glc-Rha-H]^-^, and further loss of a methyl group resulted in the fragment at *m/z* 368.08951 [M-Glc-Rha-CH_3_-H]^-^. Cleavage of the double bond in the isopentenyl group produced the fragment ion at *m/z* 313.03564 [M-Glc-Rha-C_3_H_3_O-H]^-^. On the basis of the above fragmentation patterns, it was putatively assigned as sagittasine C, an 8-C-isopentenyl-substituted flavonol bearing a 4’-methoxy group, a 3’-hydroxyl group, a rhamnosyl moiety at the C-3 position, and a glucosyl moiety at the C-7 position (**Figure 8A**). Compound 14 (t_R_ = 2.63 min) showed a quasi-molecular ion peak at *m/z* 353.0844 [M-H]^-^ in negative ion mode. Its MS/MS fragmentation produced fragment ion peaks at *m/z* 191.0542 [M-H-C_9_H_6_O_3_]^-^ (quinic acid) and *m/z* 179.0492 [M-H-C_7_H_10_O_5_]^-^ (caffeic acid). Therefore, compound 14 was putatively assigned as chlorogenic acid (**Figure 8B**).

**Figure 8:**
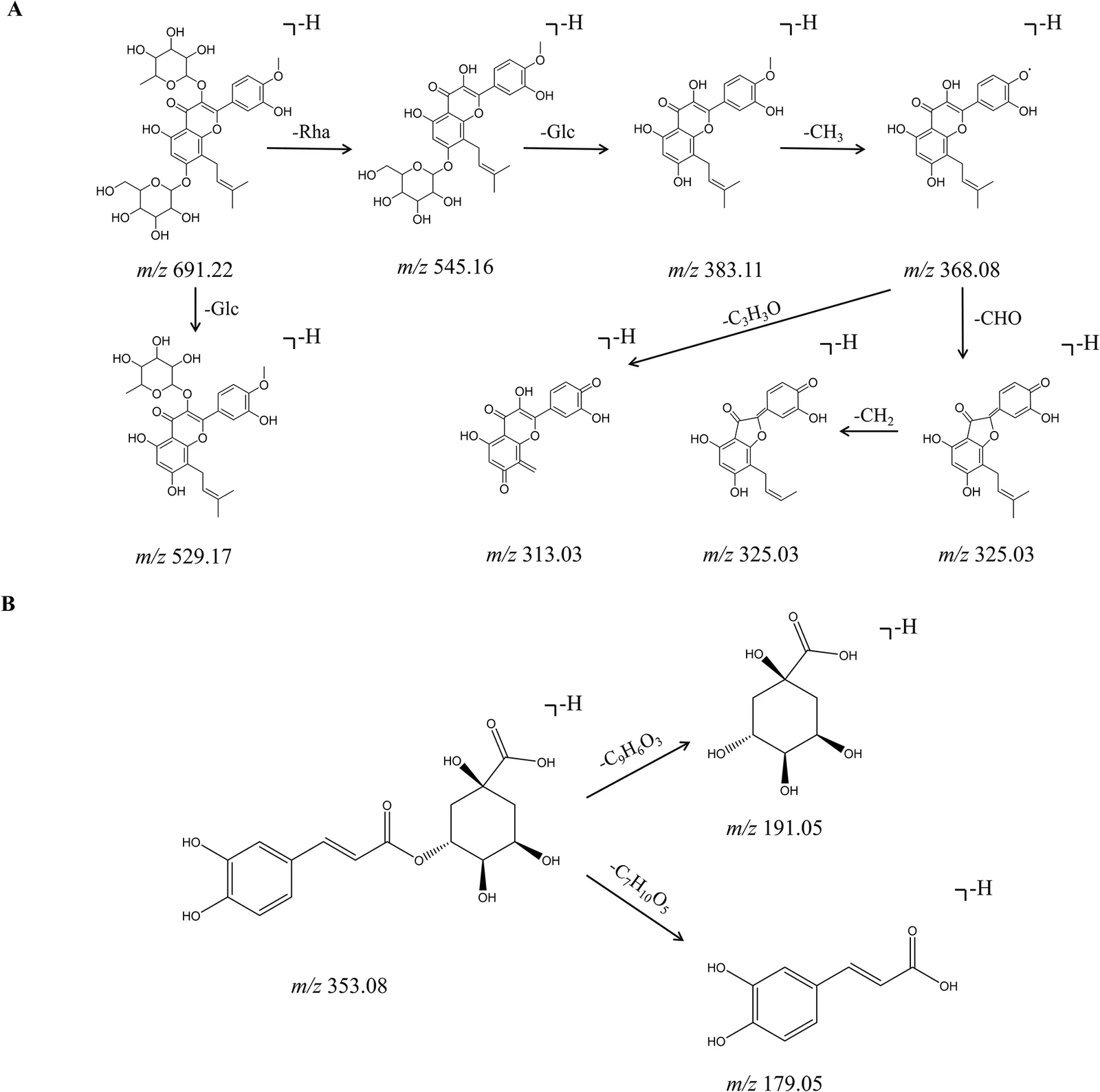
Fragmentation process of sagittasine C **(A)** and chlorogenic acid **(B)**.

### 3.8 Correlation analysis

As shown in **Figure 9**, exploratory pearson correlation analysis identified several statistically significant associations (FDR-adjusted *p* < 0.05) between antioxidant enzyme activities and secondary metabolite contents. A positive correlation was observed between antioxidant enzymes SOD and POD (*r* = 0.76, FDR-adjusted *p* < 0.05). Significant pairwise correlations were also detected between multiple key metabolites and antioxidant enzymes. Positive correlations were found between 3, 4, 2’, 4’, 6’-pentahydroxychalcone and POD (*r* = 0.72, FDR-adjusted *p* < 0.001), and between sinapic acid and SOD (*r* = 0.61, FDR-adjusted *p* < 0.05). Strong correlations were also observed among secondary metabolites: phloretin and cinnamic acid (*r* = 0.79, FDR-adjusted *p* < 0.001); phloretin and coniferyl aldehyde (*r* = 0.68, FDR-adjusted *p* < 0.01); sinapyl alcohol and coniferyl aldehyde (*r* = 0.57, FDR-adjusted *p* < 0.05); coniferyl aldehyde and cinnamic acid (*r* = 0.68, FDR-adjusted *p* < 0.01); syringin and cinnamic acid (*r* = 0.58, FDR-adjusted *p* < 0.05); syringin and tyrosine (*r* = 0.67, FDR-adjusted *p* < 0.01); syringin and homogentisate (*r* = 0.63, FDR-adjusted *p* < 0.01); syringin and ferulic acid (*r* = 0.57, FDR-adjusted *p* < 0.05); sinapoylcholine and cinnamic acid (*r* = 0.57, FDR-adjusted *p* < 0.05); sinapoylcholine and tyrosine (*r* = 0.72, FDR-adjusted *p* < 0.001); sinapoylcholine and sinapic acid (*r* = 0.65, FDR-adjusted *p* < 0.01); ferulic acid and homogentisate (*r* = 0.70, FDR-adjusted *p* < 0.01); tyrosine and homogentisate (*r* = 0.76, FDR-adjusted *p* < 0.001).

**Figure 9:**
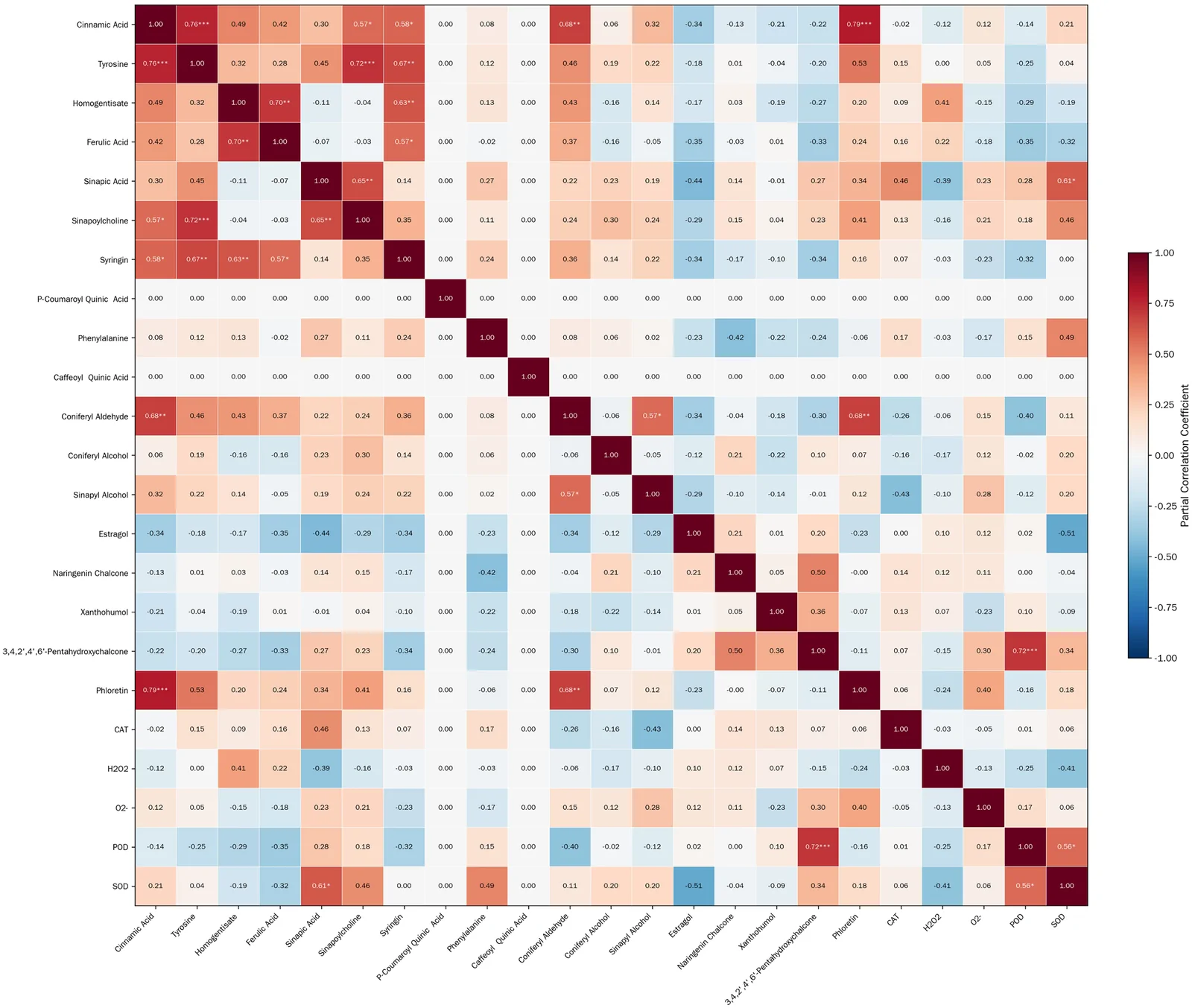
Correlation analysis of key compounds and antioxidant indices amongdifferent tissues of three *Epimedium* species. ***Correlation is significant at the 0.001 level (two-tailed). **Correlation is significant at the 0.01 level (two-tailed). *Correlation is significant at the 0.05 level (two-tailed).

## 4 Discussion

*Epimedium* species are valuable medicinal plants whose pharmacological properties are predominantly attributed to flavonoids and other secondary metabolites with antioxidant, anti-inflammatory and immunomodulatory activities (Chen et al., 2024). At present, studies on *Epimedium* species have mainly focused on the identification of bioactive constituents and their pharmacological effects (Wei et al., 2024), whereas the associations between environmental factors and secondary metabolism, as well as the interspecific and tissue-specific differences in metabolite profiles, remain to be fully characterized (Liu et al., 2025; Qin et al., 2019). By integrating meteorological monitoring, antioxidant enzyme assays, untargeted metabolomics, and correlation analysis, this study provides a foundation for the high-quality cultivation and rational utilization of *Epimedium* medicinal resources.

### 4.1 Meteorological conditions at the Epimedium sampling site

Meteorological data recorded during the study period characterized the ambient growth conditions at the *Epimedium* sampling site. Atmospheric pressure remained relatively stable, ranging from 955 to 958 hPa during 2022-2025, while temperature fluctuated mildly between 16 and 19 °C, collectively reflecting a stable climatic background generally suitable for plant growth (Wang Z. et al., 2025). The lowest annual temperatures were recorded in 2023, which may be attributable to regional climatic fluctuation and altitude variation (Liu et al., 2020). Relative humidity exhibited periodic variation between 47.9% and 91.1%, whereas precipitation showed pronounced seasonality, peaking from July to September and decreasing sharply during the winter months. These seasonal patterns are consistent with the local subtropical-temperate climatic features and match the natural growth rhythm of *Epimedium* species (Cheng et al., 2025; Terasaki Hart et al., 2025). Owing to the single-time-point sampling design, the present study cannot establish a definitive relationship between meteorological fluctuations and metabolite accumulation. Future studies with long-term, consecutive sampling are therefore needed to better clarify the potential influence of climatic factors on plant physiological characteristics.

### 4.2 Interspecific and tissue-specific differences in the antioxidant system in Epimedium species

ROS accumulation exhibited pronounced tissue- and species-specific patterns in *Epimedium* species (**Figure 2**). Within the same species, root tissues showed higher H_2_O_2_ content than leaves and stems. Roots are in direct contact with complex soil environments, where ROS accumulation is generally elevated, whereas leaf tissues generate ROS mainly through photosynthesis and UV exposure, with relatively lower ROS abundance (Gerber et al., 2025; Pasternak et al., 2023). Significant differences in H_2_O_2_ and O_2_^-^ content were also detected among the three species, revealing ROS distribution patterns across the examined germplasms (Siauciunaite et al., 2019; Cadena-Ramos et al., 2025).

Antioxidant enzyme activities also varied among tissues and species. CAT activity remained stable across all samples, suggesting its steady-state presence in *Epimedium* tissues (Raza et al., 2025). POD and SOD activities exhibited pronounced tissue specificity: higher POD activity was detected in the roots of *E. pubescens*, whereas leaf tissues of *E. wushanense* showed relatively higher SOD activity. These differences in enzyme activities corresponded to the divergent ROS distribution across organs (Schekaleva et al., 2024). The significant positive correlations between SOD/POD activities and H_2_O_2_/O_2_^-^ contents further support the coordinated operation of the antioxidant defense system in *Epimedium* species (**Figure 9**). The variation patterns of antioxidant enzyme activities were consistent with ROS levels, indicating coordinated physiological responses across *Epimedium* tissues (Nehra et al., 2025). Moreover, the antioxidant parameters varied in accordance with secondary metabolite profiles, suggesting a close association between physiological status and metabolite composition (Yang Y. et al., 2025).

### 4.3 Differential characteristics and metabolic networks of secondary metabolites in Epimedium species

Untargeted metabolomics depicted the overall metabolic composition of *Epimedium* species plants. A total of 2730 MS features were detected, among which 2268 were assigned to chemical categories at MSI Level 3. Amino acids, carbohydrates, terpene glycosides, and benzoic acid derivatives were the predominant chemical classes (**Figure 3**), in general agreement with reported chemical profiles of *Epimedium* species (Chen et al., 2025).

Correlation-based metabolic network analysis was employed to explore the co-accumulation patterns among differential metabolites, identifying 12-59 metabolites with pairwise correlations within each comparison group (**Figure 4**). Notably, several phenylpropanoid-derived compounds, including chlorogenic acid, trans-ferulic acid, plantamajoside, and osmanthuside h, were recurrently identified among the top-ranked correlated metabolites across different tissues and species. We explored the potential co-accumulation patterns of these metabolites, suggesting a potential link between phenylpropanoid and flavonoid pathways, which share upstream precursors such as phenylalanine and cinnamic acid. (**Figure 7**) (Huang et al., 2025). Likewise, carbohydrates and their derivatives (e.g., sucrose, melibiose, cellopentaose, and maltotriose) clustered within the correlation networks of roots and stems, suggesting that sugar metabolism may provide carbon skeletons for downstream secondary metabolite biosynthesis. Of particular interest, reticuline and trilinolein emerged as hub-like metabolites in multiple comparisons across tissues and species, implying their central positions linking diverse metabolic modules (Avinash et al., 2024). Moreover, several of these correlated compounds, such as chlorogenic acid and trans-ferulic acid, possess well-documented antioxidant activities, which may partly account for the potential associations observed between antioxidant enzymes and secondary metabolites (Liu et al., 2024). Nevertheless, these co-accumulation patterns are derived from statistical correlations and do not establish direct regulatory relationships; functional validation, such as pathway-specific enzyme activity measurements or targeted gene expression analysis, is required to confirm the underlying mechanisms.

KEGG pathway enrichment and MSEA were applied to characterize metabolic distribution features rather than to infer pathway regulatory status (**Figure 5** and **6**). Phenylpropanoid and flavonoid biosynthesis were highly enriched, consistent with the flavonoid-rich chemistry of *Epimedium* species (Feng et al., 2023). Multiple DAMs were mapped to the phenylpropanoid biosynthesis pathway, reinforcing the notion that interspecific and tissue-specific metabolic differences are predominantly manifested in this pathway. Carbohydrate metabolism pathways were also enriched, suggesting their potential role in providing carbon precursors for metabolite biosynthesis (Thokchom et al., 2023). Glutathione and benzenoid biosynthesis pathways exhibited differential enrichment across samples, reflecting variation in metabolic composition that may be related to the accumulation of bioactive constituents (Xu L. et al., 2025).

Enrichment patterns differed across species and tissues. Several phenylpropanoid-related metabolites were predominantly concentrated in leaf tissues, corresponding to the relatively high accumulation of bioactive constituents in leaves. The observed divergence in metabolic distribution may reflect compositional differences among the three *Epimedium* species, which could be associated with their respective growth environments and physiological traits (Li et al., 2025). The varied metabolic composition was associated with differences in antioxidant capacity, together shaping the distinct physiological and chemical characteristics of the examined germplasms (Zhao et al., 2025).

### 4.4 Correlation network between key metabolites and antioxidant enzymes in Epimedium species

Pearson correlation analysis identified exploratory associations between secondary metabolites and antioxidant enzyme activities, as well as internal metabolic connections across *Epimedium* species (**Figure 9**). Several flavonoid-related metabolites showed positive correlations with antioxidant enzyme activities. 3, 4, 2’, 4’, 6’-pentahydroxychalcone exhibited significant correlations with POD, pointing to a potential link between precursor compounds and antioxidant status (Xie et al., 2026; Xu Y. et al., 2025). However, these associations should be interpreted as exploratory, as pooled correlations across tissues and species may be confounded by between-group differences rather than true within-group biological covariation. The Metabolite-enzyme associations differed between leaf and root tissues: leaves showed combined variation of antioxidant enzymes and flavonoids, whereas roots mainly displayed potential associations between enzymes and local metabolites (Vicidomini et al., 2024).

## 5 Conclusion

This study characterized the meteorological conditions, antioxidant characteristics, and metabolomic profiles of *E. brevicornu*, *E. pubescens* and *E. wushanense*. Temperature, relative humidity, and precipitation exhibited seasonal variation, whereas atmospheric pressure remained relatively stable. Root tissues contained higher ROS levels. POD and SOD activities showed pronounced tissue- and species-specific patterns, whereas CAT activity remained stable across samples. A total of 2730 MS features were detected, and metabolic composition differed significantly among species and tissues. DAMs were mainly enriched in phenylpropanoid biosynthesis, flavonoid biosynthesis and carbohydrate metabolism pathways. 46 of these features obtained compound-level putative annotations, among which flavonoid-related compounds predominated. Potential associations were observed between antioxidant parameters and secondary metabolites. The three *Epimedium* species exhibited divergent antioxidant characteristics and metabolic profiles, with tissue- and species-specific metabolic variation mainly concentrated in phenylpropanoid- and flavonoid-related substances. These findings expand the baseline knowledge of the physiological and metabolic characteristics of *Epimedium* species and provide a foundation for further research on their quality formation.

## Data Availability

The original contributions presented in the study are included in the article/**Supplementary Material**. Further inquiries can be directed to the corresponding author.
