# Supplementary material for: Untargeted metabolomics and antioxidant responses reveal tissue- and species-specific metabolic profiles in three Epimedium species
Source: Front Plant Sci. 2026 Oct 13;17:1942146. doi: 10.3389/fpls.2026.1942146 (PMC13710339; doi:10.3389/fpls.2026.1942146)
Supplement: Supplementary file 1 [file DataSheet1.doc]

**Supplementary figures 1:** Orthogonal partial least-squares discriminant analysis (OPLS-DA) score plot of leaf sample groups. (A) and (B) OPLS-DA score plot in leaves of *E. pubescens* and *E. wushanense* in negative ion mode (left) and positive ion mode (right). (C) and (D) OPLS-DA score plot in leaves of *E. brevicornu* and *E. pubescens* in negative ion mode (left) and positive ion mode (right). (E) and (F) OPLS-DA score plot in leaves of *E. brevicornu* and *E. wushanense* in negative ion mode (left) and positive ion mode (right).

**Supplementary figures 2:** Orthogonal partial least-squares discriminant analysis (OPLS-DA) score plot of stem sample groups. (A) and (B) OPLS-DA score plot in stems of *E. pubescens* and *E. wushanense* in negative ion mode (left) and positive ion mode (right). (C) and (D) OPLS-DA score plot in stems of *E. brevicornu* and *E. pubescens* in negative ion mode (left) and positive ion mode (right). (E) and (F) OPLS-DA score plot in stems of *E. brevicornu* and *E. wushanense* in negative ion mode (left) and positive ion mode (right).

**Supplementary figures 3:** Orthogonal partial least-squares discriminant analysis (OPLS-DA) score plot of root sample groups. (A) and (B) OPLS-DA score plot in roots of *E. pubescens* and *E. wushanense* in negative ion mode (left) and positive ion mode (right). (C) and (D) OPLS-DA score plot in roots of *E. brevicornu* and *E. pubescens* in negative ion mode (left) and positive ion mode (right). (E) and (F) OPLS-DA score plot in roots of *E. brevicornu* and *E. wushanense* in negative ion mode (left) and positive ion mode (right).

**Supplementary figures 4:** PCA analyse: (A) PCA plot of *E. brevicornu* at three tissue parts. (B) PCA plot of *E. pubescens* at three tissue parts. (C) PCA plot of *E. wushanense* at three tissue parts. (D) PCA plot of leaf tissues of three *Epimedium* plant. (E) PCA plot of root tissues of three *Epimedium* plant. (F) PCA plot of stem tissues of three *Epimedium* plant. (A)~

(F) obtained the negative ion mode (left) and positive ion mode (right).

| Groups | Precursor ions | R^2^ | Q^2^ | Groups | Precursor ions | R^2^ | Q^2^ |
| --- | --- | --- | --- | --- | --- | --- | --- |
| RG_vs_WG | [M−H]⁻ | 99.5 | 93.8 | XG_vs_RG | [M−H]⁻ | 99.5 | 98.8 |
|  | [M−H]^+^ | 99.5 | 97.7 |  | [M−H]^+^ | 99.7 | 94.9 |
| Ri_vs_RG | [M−H]⁻ | 94.9 | 99.8 | XG_vs_WG | [M−H]⁻ | 99.5 | 94.5 |
|  | [M−H]^+^ | 94.9 | 99.6 |  | [M−H]^+^ | 99.8 | 94.3 |
| Ri_vs_Wi | [M−H]⁻ | 99.4 | 89 | Xi_vs_Ri | [M−H]⁻ | 99.5 | 99.3 |
|  | [M−H]^+^ | 99.4 | 80.6 |  | [M−H]^+^ | 99.9 | 90.1 |
| RY_vs_RG | [M−H]⁻ | 99.8 | 99.8 | Xi_vs_Wi | [M−H]⁻ | 99.8 | 98.3 |
|  | [M−H]^+^ | 99.8 | 99.5 |  | [M−H]^+^ | 99.9 | 85.8 |
| RY_vs_Ri | [M−H]⁻ | 97.7 | 97.6 | Xi_vs_XG | [M−H]⁻ | 99.5 | 99.8 |
|  | [M−H]^+^ | 97.7 | 98.6 |  | [M−H]^+^ | 99.5 | 99.1 |
| RY_vs_WY | [M−H]⁻ | 99.9 | 97.6 | XY_vs_RY | [M−H]⁻ | 99.9 | 95.2 |
|  | [M−H]^+^ | 99.9 | 97.4 |  | [M−H]^+^ | 99.6 | 87.4 |
| Wi_vs_WG | [M−H]⁻ | 99.8 | 99.2 | XY_vs_WY | [M−H]⁻ | 99.5 | 98.3 |
|  | [M−H]^+^ | 99.8 | 99.1 |  | [M−H]^+^ | 99.5 | 95.5 |
| WY_vs_WG | [M−H]⁻ | 99.8 | 96.9 | XY_vs_XG | [M−H]⁻ | 99.5 | 99.6 |
|  | [M−H]^+^ | 99.9 | 98.2 |  | [M−H]^+^ | 99.7 | 97.5 |
| WY_vs_Wi | [M−H]⁻ | 99.9 | 98.6 | XY_vs_Xi | [M−H]⁻ | 98.3 | 98.6 |
|  | [M−H]^+^ | 99.9 | 98.7 |  | [M−H]^+^ | 99.3 | 96.5 |

**Supplementary table 1:** Summary of validation parameters for multiple OPLS-DA models.
